# Generation of a Retargeted Oncolytic *Herpes* Virus Encoding Adenosine Deaminase for Tumor Adenosine Clearance

**DOI:** 10.3390/ijms222413521

**Published:** 2021-12-16

**Authors:** Chiara Gentile, Arianna Finizio, Guendalina Froechlich, Anna Morena D’Alise, Gabriella Cotugno, Sara Amiranda, Alfredo Nicosia, Elisa Scarselli, Nicola Zambrano, Emanuele Sasso

**Affiliations:** 1 CEINGE Biotecnologie Avanzate S.C.aR.L., Via G. Salvatore 486, 80145 Naples, Italy; chiara.gentile@unina.it (C.G.); finizioa@ceinge.unina.it (A.F.); froechlich@ceinge.unina.it (G.F.); sara.amiranda@unina.it (S.A.); nicosia@ceinge.unina.it (A.N.); nicola.zambrano@unina.it (N.Z.); 2 Dipartimento di Medicina Molecolare e Biotecnologie Mediche, Università degli Studi di Napoli Federico II, Via Pansini 5, 80131 Naples, Italy; 3 Nouscom S.R.L., Via di Castel Romano 100, 00128 Rome, Italy; m.dalise@nouscom.com (A.M.D.); g.cotugno@nouscom.com (G.C.); e.scarselli@nouscom.com (E.S.)

**Keywords:** oncolytic virus, adenosine, adenosine deaminase, targeted therapy, immunometabolism, immunotherapy

## Abstract

Background: Oncolytic viruses are immunotherapeutic agents that can be engineered to encode payloads of interest within the tumor microenvironment to enhance therapeutic efficacy. Their therapeutic potential could be limited by many avenues for immune evasion exerted by the tumor. One such is mediated by adenosine, which induces pleiotropic immunosuppression by inhibiting antitumor immune populations as well as activating tolerogenic stimuli. Adenosine is produced starting from the highly immunostimulatory ATP, which is progressively hydrolyzed to ADP and adenosine by CD39 and CD73. Cancer cells express high levels of CD39 and CD73 ectoenzymes, thus converting immunostimulatory purinergic signal of ATP into an immunosuppressive signal. For this reason, CD39, CD73 and adenosine receptors are currently investigated in clinical trials as targets for metabolic cancer immunotherapy. This is of particular relevance in the context of oncovirotherapy, as immunogenic cell death induced by oncolytic viruses causes the secretion of a high amount of ATP which is available to be quickly converted into adenosine. Methods: Here, we took advantage of adenosine deaminase enzyme that naturally converts adenosine into the corresponding inosine derivative, devoid of immunoregulatory function. We encoded ADA into an oncolytic targeted herpes virus redirected to human HER2. An engineered ADA with an ectopic signal peptide was also generated to improve enzyme secretion (ADA-SP). Results: Insertion of the expression cassette was not detrimental for viral yield and cancer cell cytotoxicity. The THV_ADA and THV_ADA-SP successfully mediated the secretion of functional ADA enzyme. In in vitro model of human monocytes THP1, this ability of THV_ADA and THV_ADA-SP resulted in the retrieval of eADO-exposed monocytes replication rate, suggesting the proficiency of the viruses in rescuing the immune function. Conclusions: Encoding ADA into oncolytic viruses revealed promising properties for preclinical exploitation.

## 1. Introduction

In recent decades, deciphering the role of immune system in the field of cancer has paved the way for the development of immunotherapy which is aimed at eliciting, or reinvigorating, innate and adaptive immune responses against tumor cells [1].

However, the local and systemic tumor immunosuppression of oncological patients is the major hurdle of immunotherapeutic treatments [2,3]. The tumor cells themselves bring into play several mechanisms to induce and maintain the tumor microenvironment (TME) in an immunosuppressive status, to inhibit the antitumor immune response and to allow the immune escape. Targeting the immune checkpoint modulators (e.g., PD-1, CTLA-4) is the most popular strategy to counteract immunosuppression and has demonstrated remarkable clinical efficacy [4,5]. Interestingly, in comparison with single immune checkpoint inhibition, combination therapy leads to improved antitumor effects compared to single immune checkpoint inhibition [3]. Although the reasons why the combined treatment improves antitumor efficacy are still debated, it is likely to rely on the synergy between different mechanisms of action (e.g., reversal of T cells anergy by anti-PD-1 combined to Treg depletion by anti-CTLA-4) [6]. This evidence suggests that targeting the right switch with pleiotropic functions could be advantageous. Currently, most of the investigational molecules target the well-known immune checkpoints (e.g., PD-1, CTLA-4, OX-40, etc.), the immunosuppressive cells (i.e., Tregs, myeloid-derived suppressor cells- MDSCs, Cancer associated fibroblast-CAF), and the production of anti-inflammatory cytokines (i.e., IL-10, TGF-β) [6,7]. Besides these well-known modulators, the immunometabolism and the purinergic pathways are emerging as promising targets in immune-related diseases and cancer therapy [8,9]. The extracellular adenosine triphosphate (eATP) and adenosine (eADO) are indeed able to modulate many facets of immune response in opposite ways. Ectopic eATP is a potent damage associated molecular pattern (DAMP) that triggers the inflammasome activation through purinergic type 2 receptors (P2X and P2Y) [10]. On the contrary, eADO has been described to inhibit immune response through G-coupled adenosine receptors (A1, A2A, A2B, and A3). Interestingly, eADO mainly results from the hydrolysis of eATP catalyzed by CD39 and CD73 ectonucleotidases, respectively, converting ATP to AMP and AMP to ADO [11,12].

Under physiological conditions, eADO plays fundamental immunosuppressive functions through receptors present on immune effector cells, thus avoiding chronic immune activation and preventing autoimmune diseases [13]. Accordingly, normal cells ensure a tight spatiotemporal control of ADO by reducing its extracellular concentration either by nucleoside transporters (ENTs) or by its degradation by ecto-adenosine deaminase (eADA) into the immunologically inactive inosine [14]. Both intracellular and extracellular ADAs have a crucial role in controlling the immune system through ADO homeostasis, as demonstrated by the development of severe combined immuno-deficiency (SCID) in patients with loss of function mutations in *ADA* gene [15].

In the hypoxic niche of cancer, Tregs, MDSCs, CAFs, and cancer cells themselves upregulate CD39/CD73 and downregulate ADA to favor eADO accumulation and immune suppression [16,17,18,19,20]. This regulation, combined with the release of a high amount of eATP as a consequence of cancer cell damage (cancer cell turnover and therapeutic agents), allows eADO to change from the physiological nanomolar concentration to micromolar into the TME [14,16,18,21,22]. As expected, CD39 and CD73 expression has been associated with poor prognosis in a variety of cancers [23]. Increasing interest in the ADO pathway is due to its pleiotropic functions including impairment of NK and T cells tumor-infiltration, proliferation, helper and cytotoxic activities, and inhibition of antigen processing and presentation [24]. On the other hand, eADO enhances the recruitment and proliferation of MDSCs and Tregs, along with the promotion of M2 polarization of monocytes/macrophages and IL-10, and VEGF secretion [25,26,27,28]. Surprisingly, adenosine receptors are also expressed by tumor cells and, upon activation, they sustain their survival, proliferation, migration, and invasiveness [14,29,30].

Inhibition of eADO and adenosinergic pathways allows the recruitment of antitumor immune populations and enhances the effector functions of the tumor-resident CD8 T cell, also by modifying the CD8 T cells/Treg ratio [31,32,33]. Small molecules and mAbs targeting CD73 and A2A/A2B receptors are currently investigated in several clinical trials, alone or in combination with immune checkpoint inhibitors (NCT02503774, NCT02754141, NCT03454451, NCT03549000) [14].

ADO pathway acquires particular relevance in the context of oncovirotherapy. This immunotherapeutic approach is based on the administration of natural or engineered viruses (oncolytic viruses-OVs) which selectively infect and kill cancer cells [34,35,36]. However, their mechanism of action relies not only on tumor cell lysis, but mainly on the immunogenic way by which cancer cells succumb. Indeed, OVs induce immunogenic cell death (ICD), which is characterized by secretion of a high amount of eATP, HMGB1, and type I IFNs, which stimulates the immune response [10,37]. Currently, many efforts are dedicated to generate novel OVs with improved ability to induce ICD and enhance ATP release [38]. Although on one hand the releasing eATP stimulates the immune response by engagement of P2 purinergic receptors (P2XRs and P2YRs), on the other hand its rapid conversion into eADO could result in a reduced efficacy of the treatment itself.

Based on this evidence, we speculated that the clearance of eADO within TME through an arming strategy could be an innovative approach to restore an anti-tumor immune competence [39].

Here, we applied this concept to HER2+ cancers, as *ERBB2* overexpression is known to characterize aggressive tumors and its interplay with eADO has also been demonstrated [40,41]. To date, across approved indications (HER2+ breast and metastatic gastric cancers), the antiHER2 mAb Trastuzumab (Herceptin) has been administered to more than two-million patients. Among the proposed mechanisms of action for Herceptin, antibody-dependent cell-mediated cytotoxicity (ADCC) is known to be inhibited by eADO as patients bearing CD39/CD73^high^ HER2+ tumors acquire resistance to Herceptin [40,41].

In light of the pivotal role of eADO in HER2+ tumors, we armed the fully virulent HER2-Targeted *Herpes* Virus (THV) R-LM113 with *Ada* cDNA to generate a novel oncolytic virus able to efficiently catabolize eADO, thus counteracting the adenosinergic pathway [42].

## 2. Results

### 2.1. Interplay between HER2 and Adenosinergic Pathways

**Figure 1:**
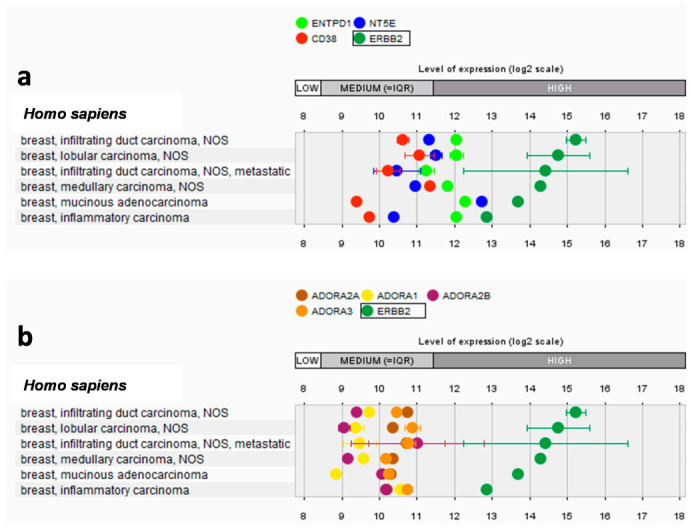
Expression of genes involved in eADO production and signaling in HER2-positive breast cancers. The magnitude of expression of ectonucleotidases involved in eADO production (*ENTPD1*, CD39; *NT5E*, CD73; CD38) (**a**) and adenosine receptors (ADORA2A, ADORA1, ADORA2B, ADORA3) (**b**) was investigated among ERBB2+ breast tumors from public transcriptomic repositories by Genevestigator.

Although targeted therapy to HER2 has proved to be effective in a plethora of cancer indications, after an initial objective response, unfortunately, many patients undergo disease progression and metastasis due to establishment of resistance [43]. This is true for both biological therapeutics (e.g., trastuzumab, CAR-T cells) and small molecules (e.g., lapatinib), underlining the need to develop novel therapeutic strategies to improve the effectiveness and duration of the responses [43]. The immunosuppressive adenosinergic pathways have been associated with the establishment of a tolerogenic niche within the tumor that hampers efficacy of targeted immunotherapy. Through RNAseq database searches, we investigated the magnitude of expression of the genes involved in adenosinergic pathway, namely, CD39 (*ENTPD1* gene), CD73 (*NT5E* gene), and CD38 (also responsible for eADO generation) among female HER2+ cancers (Figure 1a). Although a direct proportionality between HER2 and ectonucleotidases expression was not found, all the investigated tumors showed a medium to high expression of CD38, CD39, and CD73. We also investigated eADO receptors expression among HER2+ breast cancer, highlighting an expression pattern similar to that of ectonucleotidase (Figure 1b). These results provide a solid rationale for targeting eADO pathway in HER2+ cancers.

### 2.2. Generation of an Adenosine Deaminase-Armed HER2-Targeted Oncolytic Herpes Simplex Virus 1

Oncolytic viruses elicit a strong antitumor immunity and epitope spreading [34,37,44,45] by inducing cancer immunogenic cell death, which heats up the TME [46,47]. We and others recently reported that oHSV-1s elicit strong immunogenic cell death, as a high amount of eATP is released upon infection [10,48,49,50,51]. Although the release of eATP is desirable for heating up the tumor resident immune system, its conversion into eADO is to avert. OVs offer the unique opportunity to encode a payload of interest within the infected cancer cells to further improve antitumor immunity [52,53]. Retargeted Herpesviruses allow selective infection of cancer cells trough an antibody fragment targeting a given tumor antigen (e.g., HER2, MSLN, PSMA) [36,42,54,55,56]. To target the adenosine pathway in HER2+ tumors, here, we took advantage of the THV R-LM113 retargeted to human HER2 by a trastuzumab-derived scFv [42]. It has been previously armed with success, allowing the identification of insertion sites non-detrimental for viral propagation [57,58]. Adenosine deaminase enzyme (ADA) is naturally responsible for eADO clearance by its irreversible deamination to inosine whose immunosuppressive function, despite ongoing debates, is certainly milder than those exerted by eADO. Moreover, ADA expression has been recently correlated to PD-1 immune checkpoint blockade efficacy [59].

Based on this evidence, we decided to generate an HER2-retargeted oncolytic herpes virus expressing murine adenosine deaminase for intratumor clearance of eADO.

**Figure 2:**
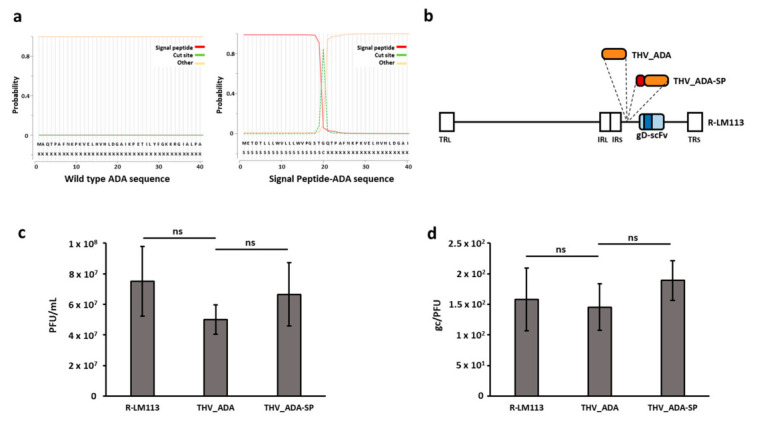
Generation of Adenosine Deaminase-armed targeted herpes viruses. (**a**) 40 N terminus aa of wild-type and SP-bearing murine ADA were analyzed by SignalP 5.0 server to evaluate the presence and proficiency of the signal peptide. Non-signal peptide (Other, yellow line) and Signal Peptide (SP, red line) moieties are reported as 0 (0%) to 1 (100%) probability. The cut site of SP (CS) is indicated as dashed green line. (**b**) Schematic representation of THVs expressing ADA and ADA-SP. The scFv targeting hHER2 (dark blue) is shown in glycoprotein D (light blue). (**c**) The yield of R-LM113, THV_ADA, and THV_ADA-SP oncolytic viruses was evaluated in SKOV3 cells infected at MOI of 0.1 pfu/cell. Yield was reported as pfu/mL obtained by plaque assay from two biological replicates of infection. (**d**) Virion infectivity was measured as gc/PFU ratio. Non-statistically significant differences were reported as Not Significant (ns).

Despite the lack of a signal peptide, adenosine deaminase is naturally secreted by specialized immune cells through a not-well-characterized pathway. We hypothesized that ectopic expression of ADA in cancer cells could result in cytosolic retention and low secretion. To improve the extracellular localization, in addition to the wild-type murine enzyme, we designed an engineered secretable layout by in-frame fusing the IgGk signal peptide (SP) upstream *mAda* coding sequence (Figure 2a). The server SignalP 5.0 (Lyngby, Denmark) was used to confirm the absence of the endogenous signal peptide and to predict the performance of the ectopic IgGk SP at the N-terminus of mADA [60].

We thus inserted an expression cassette encoding a mouse-codon-optimized murine adenosine deaminase or mADA with signal peptide (ADA-SP) into the intergenic Us1-Us2 site of an HER2-Targeted *Herpes* Virus genome (Figure 2b) [57,58]. Human CMV promoter and BGH polyA signals were used to express the transgenes of interest. The resulting BAC-genomes were transfected into HER2+ SKOV3 cells to rescue THV infectious viral particles. Single clones were isolated by plaque picking and amplified in SKOV3 cells by serial passages. Viral particles were finally purified from extracellular medium by iodixanol gradient. To assess whether ADA expression affected THV production, a comparative yield assay with unarmed R-LM113 was performed in SKOV3 cells infected at MOI of 0.1 pfu/cell. Four days after infection, the bulk-harvested intracellular and extracellular infectious viral particles were titrated by plaque assay. As depicted in Figure 2c, R-LM113, THV_ADA, and THV_ADA-SP showed comparable yields. Despite none of the known adenosine deaminase enzymes acting on DNA, we investigated if encoded ADAs could affect viral genome stability. To that end, we analyzed the integrity of expression cassette excised from viral particles at the sixth amplification passage. Sanger sequencing allowed us to confirm the insert identity and orientation (Supplementary Table S1). To further confirm that ADA enzymes do not affect genome stability, we evaluated virion infectivity and quality as genomic DNA/PFU ratio. SKOV3 cells were infected with R-LM113, THV_ADA, and THV_ADA-SP at an MOI of 0.1 pfu/cell. Infectious viral titers (PFU/mL) and genomic DNA quantification (gc/mL) in the cell lysate were, respectively, determined by plaque assay and quantitative PCR, demonstrating no significant differences between unarmed R-LM113 and ADA encoding THVs (Figure 2d).

**Figure 3:**
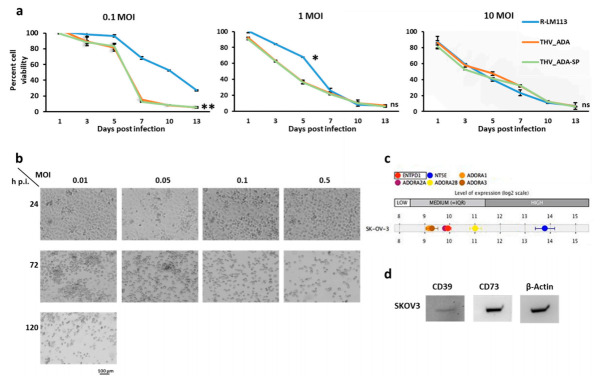
Cytotoxicity of THV_ADA and THV_ADA-SP. (**a**) The cytotoxic effect of R-LM113 (blue), THV_ADA (orange), and THV_ADA-SP (green) was assessed by Alamar Blue in SKOV3 infected at different MOI up to 13 days after infection. * and ** were, respectively, used to label *p*-value < 0.05 and <0.005. (**b**) Time-course evaluation (24, 72, 120 h post infection) by brightfield microscopy of THV_ADA-mediated cytopathic effect on SKOV3 cells infected at different MOI (0.01, 0.05, 0.1, 0.5 pfu/cell). Bar indicates 100 µm. (**c**) Transcriptomic analysis by Genevestigator of CD39, CD73, and ADO receptors in SKOV3 cells. (**d**) Expression of CD39 and CD73 in SKOV3 cells was determined by Western Blot analysis. B-Actin was used as control.

To further characterize THV_ADA and THV_ADA-SP, the cytotoxic effect on HER2+ cancer cells was compared to that of the parental R-LM113. Thus, SKOV3 cells were infected at different titers (MOI of 0.1, 1, and 10 pfu/cell) of the three viruses, and cell viability was measured up to two weeks post infection by Alamar blue assay. As expected, at high dosage (10 pfu/cell) THV_ADA, THV_ADA-SP, and R-LM113 showed the same cytolytic effect. Interestingly, at lower MOIs (0.1 and 1 pfu/cell), THV_ADA and THV_ADA-SP resulted in being even more cytotoxic than the unarmed R-LM113, presumably due to the deprivation of eADO, known to sustain cancer cell viability (Figure 3a) [14,29,30]. As Alamar blue measures cytotoxicity as inverse of metabolically active cells, in some cases viral doses did not correlate with cytotoxicity due to fast deprivation of cell substrate at high MOI (10). We further analyzed the cytotoxic effect by time-course microscopy, which confirmed a dose-dependent cytotoxicity and complete cytopathic effect even at very low MOI within 5 days post infection (0.01 pfu/cell) (Figure 3b). To support the hypothesis that eADA deprivation enhances cancer cell death, we assessed the expression of CD39, CD73, and eADO receptors (ADORAs) in SKOV3 cells. Bioinformatic analysis showed that SKOV3 cells express medium and high levels, respectively, of CD39 (*ENTPD1*) and CD73 (*NT5E*), and medium levels of eADO receptors (Figure 3c), suggesting their ability to produce and sense eADO. A2B, whose activation in tumor cells has been correlated with tumor progression, is the most expressed receptor in SKOV3 cells (Figure 3c) [30,61]. We further confirmed CD39 and CD73 expression by Western Blot analysis, in consistent agreement with bioinformatic data (Figure 3d).

### 2.3. ADA-Armed Viruses Successfully Mediate the Secretion of Functionally Active ADA in SKOV3 Cell Supernatants

**Figure 4:**
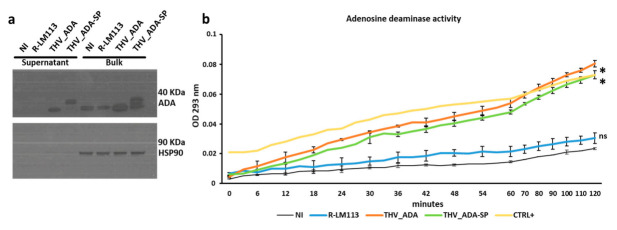
Biochemical characterization of ADA payload. (**a**) Expression of mADA was evaluated by western blot analysis in supernatants and bulk (whole cell lysate) of non-infected (NI), R-LM113, THV_ADA, THV_ADA-SP, and infected SKOV3 cells three days after infection at MOI of 0.1 pfu/cell. (**b**) Adenosine deaminase activity was measured in the supernatants described in the panel (**a**). Statistical significance was calculated between non-infected cells (NI, black line) and all the samples (R-LM113, blue line; THV_ADA, orange line; THV_ADA-SP, green line) and is reported as * for *p*-value < 0.05 and ns for not statistically significant.

To complete the in vitro characterization of THV_ADA and THV_ADA-SP, we evaluated both the expression and the enzymatic activity of ADA payloads. Supernatants and cell bulks were harvested from SKOV3 cells infected at MOI of 0.1 pfu/cell with THV_ADA, THV_ADA-SP, and unarmed R-LM113. As shown in Figure 4a, both supernatants from THV_ADA- and THV_ADA-SP-infected SKOV3 showed abundant eADA secretion. We also observed an up shift in ADA-SP molecular weight compared to wild-type ADA, likely due to the occurrence of post-translational modifications in ADA-SP hijacked to the endoplasmic reticulum/Golgi secretory pathway by SP. As expected, intracellular ADA was detected in all the samples. HSP90 was used for western blot normalization and to confirm the absence of intracellular contamination of supernatant samples (Figure 4a). Signal peptide did not result in improved secretion, presumably because the endogenous molecular machinery for ADA secretion is efficient in SKOV3 cells.

Finally, we tested the functionality of THV-encoded ADA to efficiently convert eADO into inosine. Supernatants from R-LM113, THV_ADA, and THV_ADA-SP, as well as from non-infected SKOV3 cells, were tested in ADO deamination assay. Inosine production was monitored with a kinetic measurement for up to 2 h (Figure 4b). As shown in Figure 4b, ADA encoded by THV_ADA and THV_ADA-SP converts ADO into inosine in a similar fashion to the 2000 U/mL recombinant ADA provided with the assay kit (Ctrl+). On the contrary, non-significant deamination activity was measured in supernatant from non-infected (NI) and R-LM113 sample controls. These results confirmed that THV_ADA and THV_ADA-SP were able to efficiently mediate the clearance of eADO through the expression of a functional enzyme.

### 2.4. THV_ADA and THV_ADA-SP Revert the Inhibitory Effect of Adenosine on Human Monocytes

**Figure 5:**
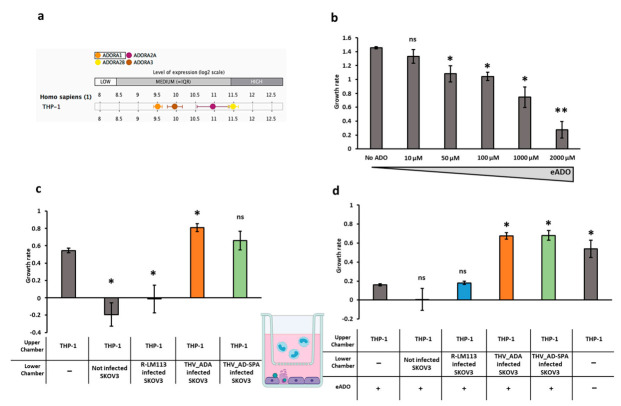
Effect of adenosine deaminase clearance on THP-1 monocytes proliferation. (**a**) RNA expression of ADO receptors was investigated in THP-1 cells by Genevestigator software. (**b**) THP-1 growth rate in medium supplemented with the indicated concentration of eADO. (**c**) Growth rate of THP-1 in SKOV3 co-culture in the presence of the indicated oncolytic virus (R-LM113, blue; THV_ADA, orange; THV_ADA-SP, green histogram). (**d**) THP-1 proliferation in THP-1/infected-SKOV3 co-culture assay in the presence of 2 mM ADO. Growth rates for (**b**–**d**) were calculated as log (2) of ratio between live THP-1 cells on day 2/day 1. Statistical significance for (**b**–**d**) were calculated in comparison to the first sample represented in the chart (No ADO; THP-1 mono-culture; THP-1 mono-culture with 2 mM ADO) and are reported as * for *p*-value < 0.05; ** for *p*-value < 0.005 and ns for not statistically significant.

THP-1 is a human leukemia monocytic cell line widely adopted in immunocytochemical analysis. Monocytes and macrophages belong to the innate immune compartment and are relevant during oncolytic virus-mediated tumor immune remodeling [34]. We adopted THP-1 cell line as a model to assess how the adenosine deaminase encoded by THV_ADA and THV_ADA-SP elicits immunomodulatory activity on an immune cell population. It has been previously demonstrated that extracellular adenosine (eADO) inhibits THP-1 proliferation [62] by signaling through ADO receptors. As previously reported, THP-1 cells showed a medium-to-high expression rate of ADO receptors (Figure 5a) [62]. We thus cultured THP-1 cells in media supplemented with increasing concentrations of adenosine (10 μM, 50 μM, 100 μM, 1000 μM, 2000 μM). As expected, eADO exerted an inhibitory effect on THP-1 replication in a dose-dependent manner, as low (10 μM), medium (50 μM and 100 μM), and high (1000 μM, 2000 μM) adenosine concentration resulted, respectively, in weak, moderate, and strong inhibition of replication (Figure 5b).

We thus setup a co-culture assay by incubating THP-1 with mock or OV-infected SKOV3 cells. We tested whether the eADO produced by CD39/CD73+ SKOV3 cells (Figure 3d) was sufficient to reduce THP-1 proliferation and whether the THV_ADA and THV_ADA-SP viruses were able to rescue THP-1 proliferation by reducing eADO concentration. In the lower chamber of a transwell, SKOV3 cells were mock-infected (PBS) or infected with R-LM113, THV_ADA, and THV_ADA-SP at MOI of 0.1 pfu/cell. THP-1 was co-cultured into the upper chamber of the transwell. THP-1 co-cultured with non-infected and R-LM113-infected SKOV3 showed a significant inhibition of replication (Figure 5c). This result underlines that the presence of tumor cells per se completely abrogates THP-1 proliferation (Figure 5c). Conversely, THP-1 co-cultured with THV_ADA- and THV_ADA-SP-infected SKOV3 cells replicated similarly or even much more than non-co-cultured THP-1 demonstrating that encoded adenosine deaminase catabolizes eADO so efficiently as to rescue THP-1 proliferation (Figure 5c).

To explore a more unfavorable condition, we further assessed whether the encoded eADA is effective in catabolizing high concentration of eADO. We replicated the THP-1/SKOV3 co-culture assay with the further addition of 2 mM eADO in the medium. As expected, in the presence of 2 mM eADO, THP-1 cultured alone or co-cultured with non-infected or R-LM113-infected SKOV3 underwent growth inhibition (Figure 5d). Conversely, despite the 2 mM eADO, the THP-1 cells co-cultured with THV_ADA- and THV_ADA-SP-infected SKOV3 replicated as well as the THP-1 cultured alone without eADO (Figure 5d). These results demonstrate that the adenosine deaminase enzymes encoded in our viruses (THV_ADA and THV_ADA-SP) can catabolize high amounts of extracellular adenosine, which are even greater than those found in the tumor microenvironment (up to 100 µM) [63].

## 3. Discussion

Cancer immunotherapy is a rapidly evolving field of oncology, with novel targets being continuously investigated. Even though classical immune checkpoint inhibitors targeting PD-1 and CTLA4 still represent the pillars of immunotherapy, innate and acquired resistance often occurs [64]. ATP and ADO are the yin and yang of the immune system as they can exert opposite effects on many immune cell populations and their functions [8]. In particular, while ATP itself can promote immune stimulation, it can undergo CD39/CD73-mediated multistep hydrolysis where it is eventually converted into immunosuppressive ADO. Multiple components of the adenosinergic pathway (in particular CD39, CD73, and A2Ar) are currently investigated in preclinical models and phase I to II clinical trials [65,66]. Oncolytic viruses represent one of the most promising combination agents to boost efficacy of immune checkpoint inhibitors [67,68,69,70]. The release of immunostimulatory molecules (e.g., ATP, HMGB1) as a consequence of immunogenic cell death represents the strength of oncovirotherapy. In this context, the fate of extracellular ATP is currently ignored. We reasoned that micromolar concentration of eATP within the TME could rapidly result in super-immunosuppressive doses of eADO.

Oncolytic viruses offer a one-of-a-kind chance to express payloads within TME by the arming approach. This delivery is highly effective and can be exploited to reduce immune-related adverse events (irAEs) caused by systemic administration and the expensive costs of recombinant proteins (e.g., cytokines, mAbs) [39,57,71]. Since targeted therapy to HER2 is highly affected by eADO, we decided to target the adenosine pathway in the frame of an HER2-targeted oncolytic herpes virus. Here, we exploited adenosine deaminase enzyme (ADA), as it is naturally responsible for eADO degradation by conversion in inosine. Both recombinant ADA protein and the viral vectored ADA gene are approved in enzyme replacement therapy (ERT) for the treatment of adenosine deaminase deficiency-based severe combined immunodeficiency (SCID), representing an excellent precedent for a favorable safety profile of the ADA enzyme [72,73]. Although the use of ADA in cancer therapy might seem obvious, only recently has a PEGylated ADA2 (PEGADA2) been investigated in preclinical cancer models revealing promising results for clinical translation [74]. While the human genome encodes two different adenosine deaminases (ADA1 and ADA2), only ADA1 orthologue has been characterized in mice. In addition, an enzymatic-independent adaptor function of ADA1 has been described. Through dual binding to both CD26 (expressed by T cells) and A2Ar (expressed by Dendritic cells), only human extracellular ADA1, but not ADA2, can bridge T and DCs, thus promoting the formation of an immunological synapse and the function of a follicular helper T cell [75,76,77,78,79,80]. Finally, it has been recently demonstrated that T cells can utilize inosine as an alternative source of carbon to glucose to sustain their functions and that inosine supplementation enhances the anti-tumor effect of ICIs [81]. Based on this evidence, we hypothesized that encoding ADA into OVs could exert pleiotropic antitumor benefits by: (i) depriving cancer cells of adenosinergic survival signaling; (ii) restoring antitumor functions of immune cell populations (T, NK, DC, APC); (iii) suppressing tolerogenic immune cells (Treg, MDSC, M2 macrophages) through eADO clearance; (iv) improving antigen presentation and Tfh cells by Tcell-to-DC crosslinking by CD26 and A2Ar [75,76,77,78,79,80]; (v) supplying intratumor inosine to reinforce antitumor T cell metabolism [81,82,83].

Here, we have described the generation of a novel HER2-targeted R-LM113 armed with murine ADA in the Us1–Us2 intergenic locus. To date, the ADA secretion mechanism is still unknown, but the absence of a signal peptide indicates that its secretion does not occur through the conventional pathway (ER/Golgi). For this reason, to ensure a massive release of ADA within the TME by non-professional cells (tumor cells), we also encoded an engineered ADA gene endowed with an ectopic signal peptide. In vitro characterization of the newly generated viruses, named THV_ADA and THV_ADA-SP, confirmed that both viruses were able to efficiently secrete a functional ADA enzyme in cell supernatants, and able to catabolize even millimolar concentration of eADO. A shift up in ADA-SP molecular weight compared to wild-type ADA was compatible with occurrence of post-translational modifications. This is in tune with the hijacking of ADA into the canonical secretion pathway (endoplasmic reticulum/Golgi) mediated by the ectopic SP. Through NetNGlyc 1.0 and NetOGlyc 4.0 servers, we identified at least three consensus sequences compatible to glycosylation (2 N-glycosylation and one potential O-linked glycosylation) [84,85].

Despite the fact that SP did not improve ADA secretion in our cell model, we speculate that it might be useful in different cancer cells less able to secrete ADA. Moreover, arming with ADA, not only is not detrimental for viral replication, but also improved cancer cell cytotoxicity, presumably through an eADO-deprivation-mechanism. Despite the fact that only free, but not DNA-incorporated, adenosine and deoxyadenosine are substrates of ADA, we confirmed that enzyme expression does not affect viral genome stability [86].

A functional assay on human monocyte derivate (THP-1 cells) confirmed that ADA and ADA-SP can catalyze eADO conversion into inosine, restoring the THP-1 growth rate. The results presented herein reveal that encoding ADA into oncolytic viruses has promising features and paves the way for preclinical translation and adoption of the ADA transgene in different THVs, as well as in all those oncolytic viruses that can host transgenes [52,53].

## 4. Materials and Methods

### 4.1. Gene-Expression Analysis of ADO Receptors and Enzymes Involved in ADO Formation

The expression of CD39 (ENTPD1 gene), CD73 (NT5E gene), CD38, ADORA2A, ADORA1, ADORA2B, and ADORA3 was analyzed by Genevestigator 9.0 software (Nebion, Zurich, Switzerland). Expression of those genes was calculated on HER2+ cancers among neoplasm of breast/female genital organs. The expression ranges (low, medium, high) were determined by considering the expression values of the entire transcriptome over all the samples included in the database. Low, medium, and high expression, respectively, correspond to the first, the interquartile range, and the fourth quartile.

### 4.2. Cell Culture, Manipulation, and Characterization

SKOV3 and THP-1 were cultured in RPMI 1640 Medium glutaMAX, at 37 °C, in 5% CO2-humidified atmosphere; the medium was supplemented with 10% heat-inactivated fetal bovine serum (FBS), 50 UI/mL penicillin, and 50 µg/mL streptomycin. Cell lines were purchased from the American Type Culture Collection (ATCC).

The expression of ADA and ADA-SP enzymes was assessed by Western Blot analysis in SKOV3 infected by THV_ADA, THV_ADA-SP, and R-LM113. The cells were infected at MOI of 0.1 pfu/cell. Post-infection supernatants (72 h) were collected and filtered by 0.2µm filters. Cell lysate was obtained by sonication. Expression of CD39 and CD73 was also assessed by western blot analysis. Lysates were prepared as previously described [87].

The samples were resolved on 4–12% SDS-PAGE gels (Invitrogen, Carlsbad, CA, USA) and transferred to membrane (Invitrogen, Carlsbad, CA, USA). Filters were probed with anti-ADA (NBP1-87404, Novusbio, Abingdon, UK), anti-CD73 (#13160 Cellsignaling, Danvers, MA, USA), and anti-CD39 (ab223842, Abcam, Cambridge, UK) antibodies in the presence of 1% milk TBS tween 0.1% for 2 h at room temperature. Anti-rabbit IgG-HRP was used as secondary antibody.

### 4.3. Oncolytic Virus Generation

Starting from the wild-type murine ADA protein sequence (Uniprot ID P03958) a mouse-codon optimization was performed using the GeneArt optimization tool (ThermoFisher, Waltham, MA, USA). The cDNA was synthesized in service and subcloned into a shuttle vector between CMV promoter and BGH polyA (ThermoFisher, Waltham, MA, USA). IgGκ signal peptide (METDTLLLWVLLLWVPGSTG) was in-frame fused to ADA to generate ADA-SP construct. The size of mADA and mADA-SP was, respectively, 1059 and 1113 bp. THV_ADA and THV_ADA-SP were generated by recombineering starting from a retargeted HSV-1_BAC carrying CMV promoter and polyA into Us1–Us2 intergenic locus as previously described [36]. Briefly, a selection cassette encoding Amp/SacB/LacZ was inserted between CMV and polyA in the first step of recombineering. Clones that resulted as positive (Blue, Amp resistance with functional SacB gene) were used for the second step of recombineering to replace the selection cassette with the transgene of interest. homology arms of 50 bp for recombineering were designed into CMV and polyA. To confirm the identity of recombinant THV_ADA and THV_ADA-SP, the expression cassette encoding ADA and ADA-SP was amplified by PCR performed on DNA extracted from rescued viral particles at P0. The amplicons were sequenced by sanger sequencing. Non-armed R-LM113 was generated from the same BAC backbone carrying CMV promoter and polyA by deleting CMV and polyA from the intergenic locus Us1–Us2. The following oligonucleotides were used to generate inserts for recombineering:

F_StepI

ctggctagcgtttaaacgggccctctagactcgagcggccgcacgccaccacccctatttgtttatttttct

R_StepI

ggcaactagaaggcacagtcgaggctgatcagcggtttaaacttaagcttttatttgttaactgttaattgtc

For_StepII_ADA

ctggctagcgtttaaacgggccctctagactcgagcggccgcacgccaccatggctcagacaccagcctt

For_StepII_ADA-SP

ctggctagcgtttaaacgggccctctagactcgagcggccgcacgccaccatggaaaccgacacactgct

Rev_StepII_ADA_ADA-SP

ggcaactagaaggcacagtcgaggctgatcagcggtttaaacttaagcttttactggtactctctgtacagcctttc

The viruses were produced and titrated in SKOV3 cells. Briefly, for viral rescue, SKOV3 cells were transfected with BAC-HSVs DNA by Lipofectamine 2000 (Life Technologies, Carlsbad, CA, USA). Single plaques were isolated and grown until full cytopathic effect (CPE) was reached in 48 mw plate. Viral particles were amplified by serial passages and were titrated by plaque assays by 10-fold scaling dilutions. Viral particles used in experiments were purified from conditioned supernatant by iodixanol gradient. For genome stability assay, viral DNA was extracted from THV_ADA and THV_ADA-SP viral particles after six serial passages by a NucleoSpin Tissue kit (740952.50, Macherey-Nagel, Duren, Germany). The entire expression cassette was extracted by PCR and sequenced by sanger sequencing (Table S1). For the infectivity assay, viral genome copies were titrated by TaqMan PCR as previously reported [36].

### 4.4. Enzymatic and Cell Viability Assays

To verify the activity of adenosine deaminase (ADA) inserted in THV_ADA and THV_ADA-SP, the Adenosine Deaminase (ADA) Activity Assay Kit was used (ab211093, Abcam, Cambridge, UK). On day 0, SKOV3 was seeded in a 12-well plate at a density of 200,000 cells/well. Cells were infected by R-LM113, and used as negative control, with THV_ADA and THV_ADA-SP at MOI of 0.1 pfu/cell. After 72 h from infection, the supernatants from each infection were collected and clarified by centrifugation at 200 rcf at 4 °C. The assay was performed according to the manufacturer’s instructions. ADA Positive Control was provided by the kit as 50U lyophilized and reconstituted in 25 μL buffer (2000 U/mL final concentration). One unit of ADA activity was defined as the amount of enzyme that hydrolyzes adenosine to yield 1.0 µmol of Inosine per minute under the assay conditions.

For cytotoxicity assay, Alamar Blue was used as previously reported [56]. Briefly, SKOV3 cells were seeded in 96-well plates at a density of 10,000 cells/well in the presence of 10% FBS RPMI medium. Cells were infected by R-LM113, THV_ADA, and THV_ADA-SP at MOI of 0.1, 1, and 10 pfu/cell; after 2 h incubation, conditioned media were replaced with 150 µL of fresh RPMI supplemented with 2.5% FBS, 50 UI/mL penicillin, and 50 μg/mL streptomycin. AlamarBlue^®^ (Biorad, Hercules, CA, USA) was added to the culture (10 μL/well) and incubated 4 h at 37 °C from day 1 to day 13 after infection. The relative cytotoxicity was expressed as the percentage difference of infected over non-infected cells.

### 4.5. THP-1 Proliferation

For THP-1 proliferation assay in co-culture with SKOV3 cells, on day -1, SKOV3 cells were seeded in 12-well plates at a density of 200,000 cells/well. On day 0, SKOV3 cells were infected with the indicated virus or mock infected. Two hours post infection, medium was replaced and THP1 was seeded at 200,000 cells/mL in the upper chamber of transwell (3460, Corning, NY, USA). Live THP-1 cells were assessed on day 1 and 2 by trypan blue coloration. The results were reported as growth rate (log base 2 cells day2/cells day1) [88]. For assay in the presence of supplemented ADO, adenosine powder (A9251, Sigma, St. Louis, MO, USA) was diluted at 2136 mg/mL in H_2_O. Four hours after seeding THP-1 cells (500,000 cells/mL), adenosine was added in the medium at different concentrations (10 µM, 50 µM, 100 µM, 1000 µM, 2000 µM). THP-1 proliferation was calculated as previously mentioned.

## Data Availability

The data and materials presented in this study are available on request.

## Supplementary Materials

The following are available online at www.mdpi.com/article/10.3390/ijms222413521/s1.
