# Supplementary material for: Generation of a Retargeted Oncolytic Herpes Virus Encoding Adenosine Deaminase for Tumor Adenosine Clearance
Source: Int J Mol Sci. 2021 Dec 16;22(24):13521. doi: 10.3390/ijms222413521 (PMC8705735; doi:10.3390/ijms222413521)
Supplement: Supplementary file 1 [file ijms-22-13521-s001.zip › ijms-1484175-supplementary.pdf]

Supplementary Table S1

Virus	Transgene	Position	Transgene sequence after six serial passages
R-LM113	-	-	-
THV_ADA	mADA	Us1-Us2	ATGGCTCAGACACCAGCCTTCAACAAGCCCAAGGTGGAACTGCATGTGCACCTGGAC<br>GGCGCCATCAAGCCTGAGACAATCCTGTACTTCGGCAAGAAGAGAGGAATCGCCCTG<br>CCTGCCGACACCGTGGAAGAACTGAGAAACATCATCGGCATGGACAAGCCCCTGAGC<br>CTGCCTGGCTTCCTGGCCAAGTTCGACTACTACATGCCTGTGATCGCCGGCTGCAGAG<br>AGGCCATCAAGAGAATCGCCTACGAGTTCGTGGAAATGAAGGCCAAAGAAGGCGTG<br>GTCTACGTCGAAGTGCGGTACAGCCCTCATCTGCTGGCCAACTCTAAGGTGGACCCC<br>ATGCCTTGGAACCAGACAGAGGGCGACGTGACACCTGACGACGTGGTGGATCTGGT<br>CAACCAGGGACTGCAAGAGGGCGAGCAGGCCTTCGGAATCAAAGTGCGGAGCATCC<br>TGTGCTGCATGAGGCACCAACCATCTTGGAGCCTGGAAGTGCTGGAACTGTGCAAGA<br>AGTACAACCAGAAAACCGTGGTGGCCATGGACCTGGCTGGCGACGAAACAATCGAG<br>GGCAGCTCTCTGTTCCCCGGCCACGTGGAAGCTTATGAGGGCGCTGTGAAGAACGGC<br>ATCCACAGAACAGTGCACGCTGGCGAAGTGGGCTCTCCTGAGGTTGTCAGAGAAGCC<br>GTGGACATCCTGAAAACCGAGAGAGTCGGCCACGGCTACCACACCATCGAGGATGAG<br>GCCCTGTACAACAGACTGCTGAAAGAAAACATGCACTTTGAAGTGTGCCCCTGGTCCA<br>GCTACCTGACAGGCGCTTGGGACCCTAAGACAACACACGCTGTCGTGCGGTTCAAGA<br>ACGACAAGGCCAACTACAGCCTGAACACAGACGACCCTCTGATCTTCAAGAGCACCCT<br>GGACACCGACTACCAGATGACCAAGAAAGACATGGGCTTCACCGAGGAAGAGTTCAA<br>GAGGCTGAACATCAACGCCGCCAAGAGCAGCTTCCTGCCTGAGGAAGAGAAGAAAG<br>AGCTGCTGGAAAGGCTGTACAGAGAGTACCAGTAA

THV_ADA-SP	mADA-SP	Us1-Us2	<u>ATGGAAACCGACACACTGCTGCTGTGGGTGCTGCTTCTTTGGGTGCCCGGATCTACAGG</u><br>CCAGACACCAGCCTTCAACAAGCCCAAGGTGGAACTGCATGTGCACCTGGACGGCGCCA<br>TCAAGCCTGAGACAATCCTGTACTTCGGCAAGAAGAGAGGAATCGCCCTGCCTGCCGAC<br>ACCGTGGAAGAACTGAGAAACATCATCGGCATGGACAAGCCCCTGAGCCTGCCTGGCTT<br>CCTGGCCAAGTTCGACTACTACATGCCTGTGATCGCCGGCTGCAGAGAGGCCATCAAGA<br>GAATCGCCTACGAGTTCGTGGAAATGAAGGCCAAAGAAGGCGTGGTCTACGTCGAAGT<br>GCGGTACAGCCCTCATCTGCTGGCCAACTCTAAGGTGGACCCCATGCCTTGGAACCAGA<br>CAGAGGGCGACGTGACACCTGACGACGTGGTGGATCTGGTCAACCAGGGACTGCAAG<br>AGGGCGAGCAGGCCTTCGGAATCAAAGTGCGGAGCATCCTGTGCTGCATGAGGCACC<br>AACCATCTTGGAGCCTGGAAGTGCTGGAACTGTGCAAGAAGTACAACCAGAAAACCG<br>TGGTGGCCATGGACCTGGCTGGCGACGAAACAATCGAGGGCAGCTCTCTGTTCCCCG<br>GCCACGTGGAAGCTTATGAGGGCGCTGTGAAGAACGGCATCCACAGAACAGTGCAC<br>GCTGGCGAAGTGGGCTCTCCTGAGGTTGTCAGAGAAGCCGTGGACATCCTGAAAACC<br>GAGAGAGTCGGCCACGGCTACCACACCATCGAGGATGAGGCCCTGTACAACAGACT<br>GCTGAAAGAAAACATGCACTTTGAAGTGTGCCCCTGGTCCAGCTACCTGACAGGCG<br>CTTGGGACCCTAAGACAACACACGCTGTCGTGCGGTTCAAGAACGACAAGGCCAA<br>CTACAGCCTGAACACAGACGACCCTCTGATCTTCAAGAGCACCCTGGACACCGAC<br>TACCAGATGACCAAGAAAGACATGGGCTTCACCGAGGAAGAGTTCAAGAGGCT<br>GAACATCAACGCCGCCAAGAGCAGCTTCCTGCCTGAGGAAGAGAAGAAAGAGCTGC<br>TGGAAAGGCTGTACAGAGAGTACCAGTAA

Table S1. Transgenes position and sequences after six serial passages. Underlined the signal peptide in ADA-SP construct
